# Applications of multimodality imaging for left atrial catheter ablation

**DOI:** 10.1093/ehjci/jeab205

**Published:** 2021-11-08

**Authors:** Caroline H Roney, Charles Sillett, John Whitaker, Jose Alonso Solis Lemus, Iain Sim, Irum Kotadia, Mark O'Neill, Steven E Williams, Steven A Niederer

**Affiliations:** 1 School of Biomedical Engineering and Imaging Sciences, King's College, London, UK; 2 Centre for Cardiovascular Science, The University of Edinburgh, Scotland, UK

**Keywords:** atria, atrial fibrillation, MRI, CT, ablation

## Abstract

Atrial arrhythmias, including atrial fibrillation and atrial flutter, may be treated through catheter ablation. The process of atrial arrhythmia catheter ablation, which includes patient selection, pre-procedural planning, intra-procedural guidance, and post-procedural assessment, is typically characterized by the use of several imaging modalities to sequentially inform key clinical decisions. Increasingly, advanced imaging modalities are processed via specialized image analysis techniques and combined with intra-procedural electrical measurements to inform treatment approaches. Here, we review the use of multimodality imaging for left atrial ablation procedures. The article first outlines how imaging modalities are routinely used in the peri-ablation period. We then describe how advanced imaging techniques may inform patient selection for ablation and ablation targets themselves. Ongoing research directions for improving catheter ablation outcomes by using imaging combined with advanced analyses for personalization of ablation targets are discussed, together with approaches for their integration in the standard clinical environment. Finally, we describe future research areas with the potential to improve catheter ablation outcomes.

## Graphical Abstract

**Figure:**
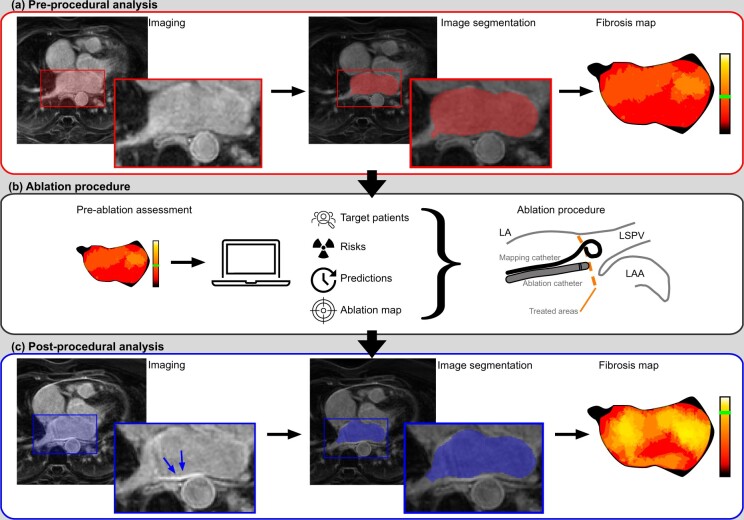

## Introduction

Atrial arrhythmias, including atrial fibrillation (AF) and atrial flutter, present a major health burden, increasing risks of stroke and heart failure and decreasing quality of life. In drug-refractory symptomatic patients with AF, catheter ablation offers an effective treatment option. However, while some patients have an excellent response to catheter ablation, many experience arrhythmia recurrence and require repeated procedures.^1^ With growing demands on clinical services, ensuring the right patients are selected and that the right therapy is delivered is of increased importance.

The process of atrial arrhythmia catheter ablation, which includes patient selection, pre-procedural planning, intra-procedural guidance, and post-procedural assessment, is typically characterized by the use of several imaging modalities to sequentially inform key clinical decisions. Increasingly, advanced pre-procedural, intra-procedural and post-procedural imaging modalities are processed via specialized image analysis techniques and combined with intra-procedural electrical measurements to inform treatment approaches.

Here, we review the use of multimodality imaging for left atrial ablation procedures. The article first outlines how imaging modalities are routinely used in the peri-ablation period (Section 1). We then describe how advanced imaging techniques may inform patient selection for ablation (Section 2) and ablation targets themselves (Section 3). Ongoing research directions for improving catheter ablation outcomes by using imaging combined with advanced analyses for personalization of ablation targets are discussed, together with approaches for their integration in the standard clinical environment. Finally, we describe future research areas with the potential to improve catheter ablation outcomes.

## Section 1: use of conventional imaging techniques in the peri-ablation period

### Patient selection using pre-procedural imaging

Pre-procedural imaging is used to assess anatomy and disease progression to help inform treatment decisions. Magnetic resonance angiography and computed tomography imaging provide detailed information on patient-specific atrial anatomy. Atrial size is the most widely accepted measures of disease progression, which can be assessed with echocardiography, magnetic resonance imaging, or computed tomography imaging.^2^ In addition, advanced imaging techniques to assess left atrial cardiomyopathy may be helpful to guide patient selection (see Section 2).

Pre-procedural imaging is routinely used to exclude the presence of left atrial thrombus prior to catheter ablation.^3^ A variety of imaging modalities have been used for this purpose, including transoesophageal echocardiography and cardiac computed tomography.^4–7^ Intra-cardiac echocardiography may also be adequate for this indication.^8^ Pre-procedural cross-sectional imaging has the additional benefit of providing information on left atrial and pulmonary vein size and anatomy,^9^^,^^10^ and magnetic resonance imaging and echocardiography provide additional and important assessments of left ventricular systolic function.^11^

### Intra-procedural imaging

Traditionally, fluoroscopic imaging has been used to guide AF ablation. Given the exposure to ionizing radiation consequent upon fluoroscopic imaging, some centres have reported ‘zero fluoroscopy’ procedures.^12^ Fluoroscopic ionizing radiation exposure during AF ablation has decreased significantly with the evolution of electroanatomic mapping systems.^13–20^ Intra-procedural ultrasound imaging (either intra-cardiac echocardiography or transoesophageal echocardiography) is commonly used as an adjunctive intraprocedural imaging modality to facilitate trans-septal puncture.

Interventional magnetic resonance imaging is a developing field and electrophysiology procedures under magnetic resonance guidance have previously been reported.^21^^,^^22^ Interventional magnetic resonance imaging offers an alternative radiation-free approach to catheter ablation together with the potential benefits of direct intra-procedural substrate visualization,^23^ although AF ablation under interventional magnetic resonance imaging guidance has not been reported at present.

### Post-procedural imaging

Before discharge and during patient follow-up, imaging is used for patient monitoring and the identification of complications. Echocardiography may be used to identify the presence of pericardial effusion.^24^ If an atrio-oesophageal fistula is suspected, computed tomography with contrast may be used for diagnosis.^25^ Pulmonary vein stenosis following pulmonary isolation may be under-diagnosed.^26^ Computed tomography or magnetic resonance angiography can be used to detect pulmonary vein stenosis with comparable accuracy to invasive angiography.^27–29^ Late-gadolinium enhancement cardiac magnetic resonance (LGE-CMR) imaging has been reported to assess atrial ablation lesion formation,^30^^,^^31^ although the sensitivity of existing LGE-CMR for detecting gaps in ablation lesion sets is debated.^32^

## Section 2: using advanced imaging techniques to inform patient selection for ablation

Patient selection for ablation therapy involves detailed consultation between patient and physician, considering the clinical features of the arrhythmia, patient co-morbidities, complications of AF, patient preferences, and prior treatment responses. Some of these factors have been encapsulated within clinical scoring systems which provide additional quantification of the likely outcome of AF ablation. For example, the CHA_2_DS_2_VASc, CHADS_2_, and R_2_CHADS_2_ scores have been linked to arrhythmia recurrence after single procedure AF ablation, although the predictive value is modest. Scoring systems specific for AF recurrence following ablation have also been developed including the DR-FLASH score (comprising diabetes mellitus, renal dysfunction, persistent form of AF, left atrial diameter >45 mm, age >65 years, female sex, and hypertension),^33^ and the CAAP-AF score (coronary disease, atrial diameter, age, persistent or long-standing AF, number of anti-arrhythmic drugs failed, and female sex).^34^ The performance of these scores is modest, with *C*-statistics of 0.767 and 0.650, respectively.

One contributing factor for the modest performance of such scoring systems may be their dependence upon the detection of factors which indirectly influence atrial electropathophysiology. In this context, advanced imaging techniques could provide direct measures of atrial structure and function that may provide additional value for predicting response to therapy and therefore hold utility for informing patient selection for ablation. One of the simplest metrics to calculate is atrial volume, and Costa *et al.*^2^ demonstrated that atrial volume is more important than AF type for predicting whether AF will recur following pulmonary vein isolation across a cohort of 809 patients. Using more advanced analyses, LGE-CMR and adipose tissue computed tomography imaging have been reported to provide information on disease progression and likelihood of AF recurrence following catheter ablation.^35^^,36^

Pre-procedural LGE-CMR imaging has been used for the quantification of atrial fibrosis, which changes with disease progression. There are multiple software platforms available for processing atrial LGE-CMR including Cemrgapp^37^ (cemrgapp.com), ADAS3D Medical (adas3d.com), Merisight Inc. (http://merisight.com/), and Music (https://www.ihu-liryc.fr/en/music/). Many of these platforms are proprietary, which makes direct comparisons of methods challenging. However, intra-observer reproducibility of the open-source Cemrgapp has been demonstrated.^38^ In general, LGE-CMR images are interpreted by classifying regions of the tissue as fibrotic. Voxel intensities are first transformed to either an image intensity ratio or number of standard deviations from the average blood pool intensity. A threshold is then applied to classify the tissue as fibrotic. Different centres have performed studies to verify their choice of threshold for identifying atrial fibrotic tissue from LGE-CMR scans, either through comparing to healthy volunteers,^39^ through comparison with bipolar peak-to-peak voltage,^40^ or through comparison with ablation scar histology.^41^ Benito *et al.*^42^ provide a comprehensive review of using LGE-CMR for assessing fibrosis.

Atrial shape measurements may indicate likelihood of AF recurrence. For example, Bieging *et al.*^43^ demonstrated that a more round left atrial shape, as well as a shorter and more laterally rotated appendage was predictive of recurrence.

Following quantification of atrial fibrosis from LGE-CMR imaging, Khurram *et al.*^36^ showed that patients with a higher degree of atrial fibrosis have a higher rate of AF recurrence across a cohort of 165 patients. Similarly, the Delayed-Enhancement MRI Determinant of Successful Radiofrequency Catheter Ablation of Atrial Fibrillation (DECAAF) clinical trial indicated that the degree of atrial late-gadolinium enhancement was independently associated with AF recurrence following catheter ablation in a cohort of 260 patients.^44^ Notably, these findings have been confirmed by some studies but also refuted by other studies.

Recent studies use computed tomography to quantify adipose tissue content, which has been shown to affect AF maintenance mechanisms. For example, in a mechanistic study, Nalliah *et al.*^45^ showed that higher adipose tissue content is correlated with increased fibrosis, slower conduction, higher degrees of electrogram fractionation, and increased lateralization of connexin40 gap junctional protein. In a clinical study, El Mahdiui *et al.*^35^ demonstrated that posterior left atrial adipose tissue attenuation is predictive of AF recurrence following catheter ablation therapy. Similarly, a meta-analysis of 12 studies found that total epicardial fat tissue volume and thickness seem to be associated with AF recurrence following catheter ablation therapy.^46^

## Section 3: using advanced imaging techniques to guide ablation procedures

### Electroanatomic mapping: an intra-procedural imaging technique

Electroanatomic mapping is usually considered to be distinct from medical imaging, but it holds many similarities to medical imaging techniques by providing both anatomical and structural cardiac information. Electroanatomic mapping systems use a variety of technologies to locate catheters within the intracardiac cavities or pericardial spaces to create a 3D anatomical representation (‘image’) of the cardiac chambers.^15^ Electrical recordings from the same catheters provide information on activation rates,^47^ conduction patterns,^48^ conduction speed,^49^ wall thickness,^50^ and voltage.^51^ Derived electrical measures—for example dominant frequency, local activation time, bipolar peak-to-peak voltage, and electrogram fractionation^52^—can be displayed as scalar fields on the chamber shell image.

Electroanatomic mapping is used routinely for guiding atrial arrhythmia ablation however, as with all medical investigations, it has several known limitations. First, determining disease progression from electroanatomic mapping data is challenging and an area of active research. For example, electroanatomic voltage maps are often used as a surrogate indicator for the presence of fibrosis, yet voltage values depend on wavefront direction,^48^ electrode size and contact,^53^ pacing frequency,^54^ and atrial rhythm.^55^ This makes it challenging to interpret maps of voltage amplitude for characterizing atrial fibrosis. Secondly, it is challenging to record electrograms with an even spatial distribution and global coverage of the entire atria. This means there is a degree of uncertainty in the electrical properties of some areas of the atrial tissue and useful information may be missed.^56^ Thirdly, it is challenging in general to use electrogram metrics to guide ablation.^57^ While many metrics have been proposed, none of the metrics have been unambiguously successfully evaluated in large clinical trials to guide ablation procedures.^58^

Augmenting electroanatomic mapping with imaging data has the potential to overcome some of these challenges and further contribute to the guidance of ablation procedures. In the following sections, we detail how magnetic resonance imaging, computed tomography and rotational angiography data have been used during catheter ablation therapies to inform ablation approaches.

### Image integration with magnetic resonance and computed tomography imaging

Pre-procedure magnetic resonance imaging and computed tomography provide high-contrast and high-resolution images that allow a complete description of the patient’s atrial anatomy. These images can be used to create an atrial anatomical shell that can be registered with the anatomy derived from electroanatomic mapping systems.^15^ Performing image integration in this way to combine pre-procedural anatomical data from imaging with electroanatomic mapping data may reduce fluoroscopy times^13–20^ and procedure times.^16^^,^^17^^,^^59^ Some studies have found improved clinical outcomes following ablation with image integration,^18^^,^^60–62^ while others have found no difference in outcomes,^13^^,^^14^^,^^16^^,^^63^^,^^64^ which is supported by meta-analysis.^65^ A major challenge in image integration remains registration errors between modalities. Registration errors depend on the size of the atria^66^ and the period of the atrial contraction cycle when the image was acquired,^67^ but appear to be independent of the presenting rhythm.^68^ These potential confounding factors may explain why some studies have found the accuracy of image integration to be insufficient to guide ablation procedures,^69^^,70^ while others have reported improved clinical outcomes with image integration.^18^^,^^60^^,^^62^^,^^71^

### Oesophageal location

Damage to the oesophagus during AF ablation carries a risk of atrial-oesophageal fistula which is amongst the most devastating complications of AF ablation.^72^^,^^73^ The oesophagus can be readily identified on pre-procedural computed tomography and cardiac magnetic resonance imaging and merged with the electroanatomic mapping system geometry. Scazzuso *et al.* demonstrated a good agreement of oesophageal position between computed tomography and fusion imaging when computed tomography imaging was within 48 h (83.3% vs. 64% for non-recent computed tomography imaging). They reported that knowledge of oesophageal location modified their ablation approach in 51% of cases.^74^ In contrast, Daoud *et al.*^75^ compared computed tomography images taken 1 week pre-procedure with intraprocedural contrast oesophagram and concluded that computed tomography did not reliably detect the location of the oesophagus. The oesophagus is mobile within the thorax and hence its position on pre-procedural imaging may not represent its position during the ablation procedure; however, identification of its course may be beneficial in planning ablation strategy if confirmed with other intra-procedural modalities.

### Late-gadolinium enhancement cardiac magnetic resonance imaging

In addition to its potential use in patient selection for ablation outlined earlier, LGE-CMR imaging has also been investigated for ablation procedure guidance. Two general areas have been evaluated: identifying ablation targets using LGE-CMR and evaluating ablation scar following ablation.

### Identifying ablation targets using LGE-CMR

It is hypothesized that fibrotic areas represent a potential ablation target since fibrosis slows atrial conduction and alters atrial electrophysiology, which might anchor re-entry.^76^ Indeed, areas of fibrotic tissue identified via electroanatomic voltage mapping have been targeted for ablation, for example, the box isolation of fibrotic regions approach, with some success.^77^ During AF ablation, atrial anatomy with fibrotic labelled regions can be registered to the electroanatomic mapping data to facilitate intra-procedural ablation guidance based on LGE-CMR alone or LGE-CMR as an adjunct to conventional electrophysiological data.

The use of LGE-CMR to guide the index ablation strategy has been investigated. The Magnetic Resonance Imaging-Guided Fibrosis Ablation for the Treatment of Atrial Fibrillation (ALICIA) trial compared MRI-guided fibrosis ablation with pulmonary vein isolation to pulmonary vein isolation alone across 155 patients, with a primary endpoint of rate of recurrence at 12 months, but found that ablating atrial fibrosis detected using LGE-CMR with pulmonary vein isolation was not more effective than pulmonary vein isolation alone.^78^ The patient population in ALICIA consisted of both paroxysmal and persistent AF and had an overall low fibrosis burden. In contrast, the Efficacy of Delayed Enhancement MRI-Guided Ablation vs. Conventional Catheter Ablation of Atrial Fibrillation (DECAAFII) trial is investigating whether ablation that targets areas of high LGE-CMR intensity is superior to pulmonary vein isolation in persistent AF patients expected to have a higher fibrosis burden. The results of DECAFF II are awaited.

Using an alternative image analysis approach, Kiuchi *et al.* also demonstrated proof-of-concept for identifying ablation targets using LGE-CMR imaging. In their study, only regions of ‘fragmented LGE areas’ were ablated, based on prior simulation studies indicating that these regions are critical for anchoring meandering AF drivers. In a non-randomized study, AF organization and termination was demonstrated in a cohort of 31 persistent AF patients with a low rate of arrhythmia recurrence during follow-up in this group.^79^

The use of LGE-CMR for informing the approach to ablation of recurrent atrial arrhythmia following index catheter ablation has also recently been studied in a population of patients in whom the mode of recurrence was AF in 46 patients and atrial tachycardia in 56 patients. Patients with recurrent AF were treated with a fibrosis homogenization approach, whilst, in those with recurrent atrial tachycardia, ‘dechanneling’ of LGE-CMR detected isthmi was studied.^80^ In this later group, approximately half of patients were treated with ablation guided by established electrophysiological techniques whereas the remainder were treated dependent upon the LGE-CMR findings. Notably, the subsequent treatment response was similar (64–67% freedom from arrhythmia) across the three groups of patients thus studied (recurrent AF treated with LGE-CMR homogenization, recurrent atrial tachycardia treated with conventional approach and recurrent atrial tachycardia treated with LGE-CMR-guided dechanneling). As already commented,^81^ although this study suggests the feasibility of a new approach for ablation of recurrent atrial arrhythmias, further studies are needed to confirm the imaging features which best identify appropriate ablation targets. Nevertheless, even in the setting of similar clinical results, there are clear advantages of the utility of pre-procedure treatment planning based on non-invasive imaging data.

### Evaluating ablation scar following ablation

Assessing the degree and distribution of atrial LGE intensities following index ablation could have a role in planning repeat ablation approaches. For example, identifying pulmonary vein reconnection in a patient with AF recurrence following ablation would identify a clear ablation target which can be useful in discussing repeat ablation. Quinto *et al.*^82^ reported that LGE-CMR can be used to identify anatomical venoatrial gaps prior to repeat pulmonary vein isolation procedures. Using this approach, they demonstrated shorter procedures and better clinical outcomes for a case-control study of 35 patients. Although other studies have also reported accurate identification of the site of pulmonary vein reconnection using post-ablation LGE-CMR imaging,^30^^,^^31^ this finding has not been confirmed by other studies.^32^

**Figure 1:**
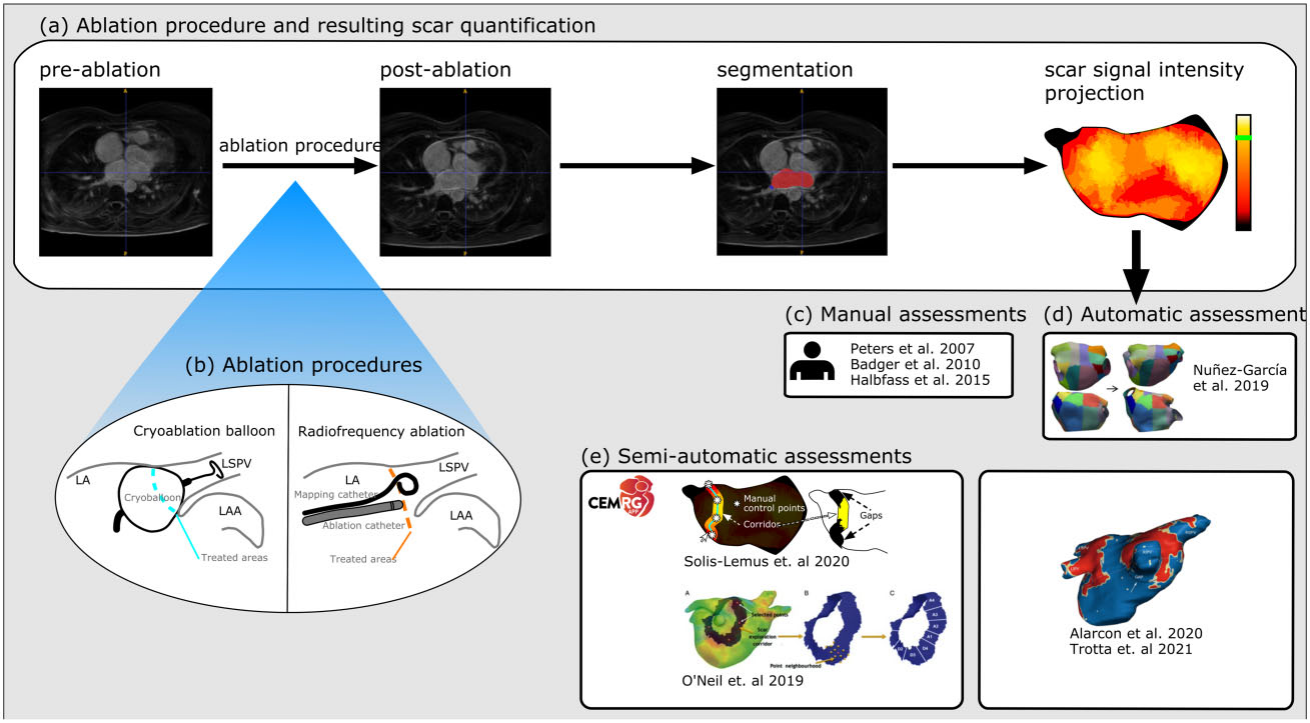
Representation of post-ablation scar quantification and methods for assessing pulmonary vein isolation. (*A*) LGE-CMR scans corresponding to pre- and post-ablation. The scar is commonly assessed by segmenting the image, creating a surface mesh of the segmentation, and performing a maximum intensity projection of the intensity near the atrial wall. (*B*) Simplified diagrams of the ablation procedures for pulmonary vein isolation. The methods to assess pulmonary vein isolation appear in (*C*–*E*). (*C*) Methods that require full manual intervention. (*D*) The fully automated method by Nunez-Garcia *et al.*,^86^ which depends on a very specific parcellation of the atrium. (*E*) Semi-automated approaches. LGE-CMR, late-gadolinium enhancement cardiac magnetic resonance.

Part of the reason for this variability in results may be the availability of few methods to robustly assess ablation lesion contiguity using LGE-CMR. Indeed, in many cases, visual inspection rather than quantitative analysis has been used.^83–85^ In Nuñez Garcia *et al.*,^86^ a fully automated approach to quantify the ablation was presented. However, the variability in pulmonary vein morphology limits this approach to a configuration of four pulmonary veins, as the method requires a very specific parcellation of the atrium. In Solís-Lemus *et al.*,^87^ a set of semi-automatic methods, built on top of CemrgApp software, were presented where users could compare pre- and post-ablation scans in two ways: comparison of registered shells’ scars, and user-defined ablation corridors with gap measurements. These methods are demonstrated in *Figure 1*. The evolution of these tools may help to improve the reproducibility of post-ablation LGE-CMR scar quantification.

LGE-CMR has also been used as a research modality to quantify the effects of different technologies for left atrial ablation. For example, Alarcón *et al.*^88^ and Trotta *et al.*^89^ utilized ADAS-AF software to compare cryoballoon and radiofrequency ablation without finding significant differences between the ablation techniques. Similarly, O’Neill *et al.*^90^ compared different techniques for radiofrequency ablation and demonstrated that Ablation Index-guided point-by-point ablation resulted in a lower scar burden and width with more complete pulmonary vein encirclement than a conventional drag lesion approach. These studies demonstrate that post-ablation LGE-CMR might be used to help inform general approaches to ablation rather than the patient-specific approaches outlined above.

### Computed tomography imaging

As outlined earlier, computed tomography quantification of epicardial adipose tissue has been investigated for the prediction of arrhythmia recurrence following ablation. In addition, given the potential effects of adipose tissue on atrial electrophysiology, computed tomography quantification of epicardial adipose tissue has also been used to target ablation. In a single study, Nakahara *et al.* performed pulmonary vein isolation followed by epicardial adipose tissue ablation in a cohort of 60 persistent AF patients. This study found that this ablation approach eliminated high frequency sources and led to a 78% freedom from AF on antiarrhythmic drugs at 16-month follow-up.^91^ This response rate was significantly greater than an historical control group however it should be noted that the control group underwent stepwise ablation including complex fractionated ablation which has not been shown to be superior to pulmonary vein isolation alone in randomized trials.^1^ Further data are therefore needed, ideally in the form of randomized trials, to fully elucidate the role of computed tomography imaging as an adjunct for guiding ablation.

**Figure 2:**
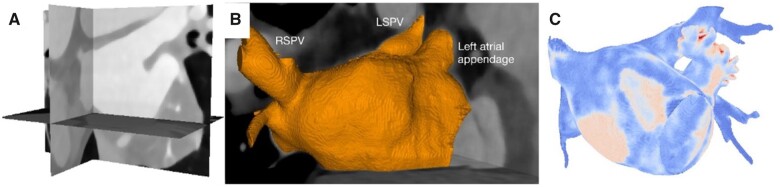
Multiple advantages of pre-procedural contrast-enhanced computed tomography imaging to inform atrial fibrillation ablation. (*A*) Contrast-enhanced computed tomography imaging, which is routinely and widely performed to exclude left atrial appendage thrombus prior to left atrial ablation. (*B*) 3D segmentation of left atrial blood pool, which may be rapidly acquired from contrast-enhanced computed tomography imaging using open-source software or proprietary programs within the commercially available electroanatomic mapping systems. Computed tomography-derived left atrial anatomy provides structural information about left atrial appendage size, shape and the configuration of pulmonary veins which may be helpful during an atrial fibrillation ablation procedure. (*C*) Experimental use of contrast-enhanced computed tomography imaging to calculate a global left atrial wall thickness map. Computed tomography-derived tissue thickness may provide additional substrate information.

Pre-procedural contrast-enhanced computed tomography data can also be post-processed to assess atrial wall thickness. Whitaker *et al.*^92^ provide a comprehensive review of the role of left atrial wall thickness in atrial arrhythmias. Both Wang *et al.*^93^ and Mulder *et al.*^94^ showed that local atrial wall thickness is associated with acute pulmonary vein reconnection after ablation index-guided pulmonary vein isolation. This provides data that support the intuitive idea that local wall thickness may be an important determinant of transmural ablation and durable pulmonary vein isolation. An example of a proposed computed tomography processing technique to derive tissue thickness is shown in *Figure 2*. Future strategies titrating ablation energy delivery dependent upon pre-procedural assessment of atrial wall thickness, or intra-procedural assessment of wall thickness using dielectric imaging, may be able to optimize ablation energy delivery to optimally balance efficacy and safety.^50^

### Rotational angiography

Registration of imaging and electroanatomic mapping data can be challenging because cross-sectional imaging is typically performed prior to the ablation procedure, and so the patient’s rhythm and volume status may be different, the patient’s position in the imaging scanners may be different to their position during the ablation procedure and there are inherent limitations in the anatomical accuracy of electroanatomic mapping systems. Rotational angiography overcomes this limitation to produce less registration challenges by imaging the left atrium during the procedure.^95^ Rotational angiography uses the catheter laboratory fluoroscopy system to collect images by rotating the C-arm around the patient in a 240° arc over 4 s.^96^ 3D rotational angiography can quickly obtain detailed left atrial and pulmonary vein anatomical information. Rotational angiography can be used instead of conventional computed tomography or magnetic resonance imaging or can be overlaid on 2D fluoroscopy images. Carpen *et al.*^16^ showed that using rotational angiography for 3D reconstruction and fusing this with electroanatomic mapping data (NavX fusion) may lead to reduced procedure times and radiation exposure compared with using electroanatomic mapping data alone.

### Future perspectives

Successful integration of data from imaging and electroanatomic mapping to guide ablation therapy requires an understanding of the relationship between information measured using each modality. The application of imaging techniques to guide left atrial ablation procedures is however a rapidly changing field with new insights arising from both new imaging techniques and new analysis techniques including biophysical simulations and machine learning. Here, we outline some of the recent advances that are not part of the standard clinical workflow but may provide useful information for informing ablation treatment decisions in the future.

### Imaging technology developments

New imaging developments include magnetic resonance imaging techniques to allow simultaneous visualization and quantification of both fibrosis and epicardial adipose tissue^97^; new magnetic resonance image navigator techniques which have the potential to reduce artefacts and improve the identification of left atrial fibrosis^98^; new positron emission tomography tracers for imaging the cardiac autonomic nervous system^99^; and new positron emission tomography tracers with the potential to revolutionize the identification of native cardiac fibrosis in both the ventricle and atria.^100^ Together, these and other techniques have the potential to reveal unprecedented levels of detail of left atrial anatomy, structure and function, and collect new information which may become central in guiding treatment decisions for AF patients in the future.

Alongside these developments, the evolution of image analysis techniques is continuing at speed. The increasing availability of public cross-sectional imaging datasets now facilitates the intra-group comparison of techniques for image segmentation or registration across large datasets from different centres and scanner vendors.^101^ Contributing to reproducibility, there are several open-source software platforms (for example, CemrgApp^37^ and OpenEP^102^) for processing imaging and electroanatomic data sets. Releasing codes and trained networks to the community will advance the field and enable reproducible operator-independent analyses.

### Using strain and measures of atrial mechanics to guide therapy

**Figure 3:**
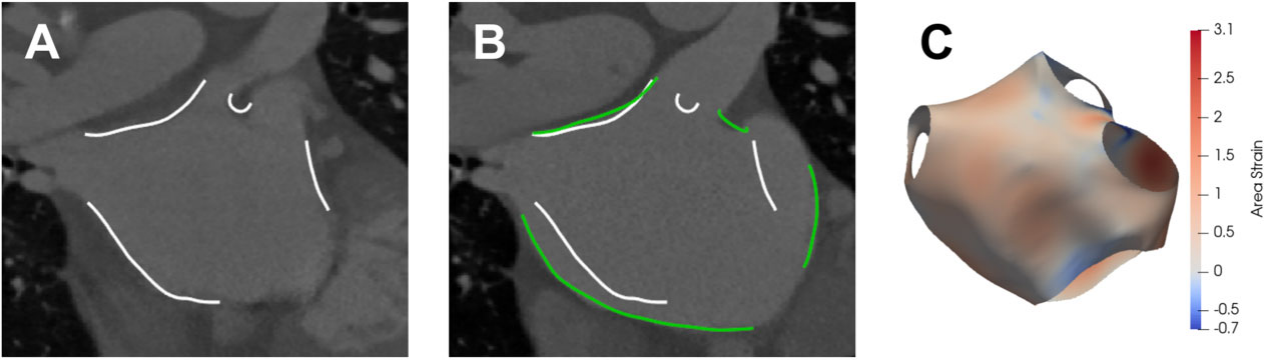
Registration of the left atrial endocardial body from end-diastole to end-systole. The white reference contour of the left atrial body at end-diastole is overlaid upon the end-diastole frame in (*A*). The green contour of the left body at end-systole is overlaid upon the end-systole frame in (*B*), and the end-diastole left atrial contour is shown to demonstrate left atrial motion from end-diastole to end-systole. (*C*) The area strain map of the left endocardial surface from end-diastole to end-systole.

Assessment of left atrial mechanics may add diagnostic and prognostic value to the management of AF patients. Fibrotic changes in the atrial wall that sustain and are promoted by AF inhibit local atrial mechanics by disrupting tissue conductivity and cellular organization.^103–105^ The decreased myocardial contractility and increased stiffness in fibrotic regions directly impacts the local atrial mechanics. There is evidence that atrial fibrosis identified by LGE-CMR is related to the strain and strain rate derived from echocardiography techniques.^106^ Greater extent of fibrosis was found to be associated with lower left atrial strain and strain rate values, and these mechanical indices have been suggested to provide additional information on AF burden. Strain analysis is most commonly conducted using echocardiography techniques, such as tissue Doppler imaging and speckle tracking echocardiography. In addition, cardiac magnetic resonance imaging using tissue tagging^107^ and feature tracking techniques, or contrast-enhanced retrospective gated computed tomography imaging using feature tracking have been proposed as high-resolution and 3D imaging modalities to measure atrial strain. *Figure 3* shows results of recent studies to optimize conventional registration methods to track the left atrial endocardium using retrospective gated computed tomography imaging.^108^ 3D strain imaging could provide new markers for identifying fibrotic regions and guiding ablation.

### Using anatomical biophysical models to inform ablation therapy

Exciting new advances in patient-specific biophysical modelling based on imaging data mean that biophysical models constructed from pre-procedural imaging data, and potentially calibrated to in-procedure electrical data, may be used in the future to guide ablation therapy. The OPTIMA trial is a current clinical trial at Johns Hopkins University that will compare pulmonary vein isolation to pulmonary vein isolation plus ablation of targets identified through biophysical simulations. Preliminary data for 10 persistent AF patients with atrial fibrotic remodelling suggest that biophysical simulations based on LGE-CMR data might be used to identify regions of fibrotic tissue that sustain AF.^109^ Like the ablation approaches that target LGE-CMR from imaging alone, this technique is more likely to be appropriate for patients with significant atrial fibrotic remodelling.

Anatomical biophysical models derived from imaging data may also be used to evaluate different ablation or antiarrhythmic drug therapies through virtual clinical trials.^110–113^ By performing biophysical simulations across populations, it may be possible to investigate how antiarrhythmic drug and ablation therapy efficacy depends on anatomical properties, as well as the degree and distribution of fibrotic remodelling. Through the use of biophysical simulations, it may also be possible to develop new metrics from routinely available clinical data which can be readily applied in the clinic. For example, we previously combined the surface area of the left atrium (a metric readily available through cross-sectional imaging) with electrical measures of atrial conduction speed and effective refractory period to estimate left atrial effective conducting size. Using biophysical simulations, we showed how this metric could subsequently be used to select ablation strategy on a patient-specific basis.^114^

Future research directions for biophysical simulation studies include developing techniques for fast calibration to electroanatomic mapping data^115^^,116^ and fast simulation techniques^117^^,118^ so that biophysical simulations incorporating electrical data measured during a procedure can be used to inform the ablation approach. We also expect that future research directions will interpret local electrical or imaging metrics within the wider context of the patient’s demographics, through machine learning approaches, or within the context of physiology encoded in biophysical models.^119^

### Using imaging data and machine learning to predict ablation outcome

Machine learning and statistical techniques may be used across populations of patients to predict, from imaging data, how likely it is that AF will recur after catheter ablation therapy. Bratt *et al.*^120^ found that atrial volume is an independent predictor of AF, where volume was calculated from computed tomography scans that were automatically segmented using deep learning approaches. Varela *et al.*^121^ built a statistical shape model from 144 AF magnetic resonance angiography images and showed that using vertical asymmetry together with left atrial sphericity is predictive of AF recurrence. Firouznia *et al.*^122^ calculated fractal-based metrics for the left atrium and pulmonary veins from computed tomography data for 203 patients pre-ablation and trained machine learning classifiers to demonstrate association with likelihood of post-ablation AF recurrence. In contrast, Ebersberger *et al.*^123^ found no relation between anatomical metrics derived from computed tomography and early AF recurrence at 3–4 months post-ablation.

Machine learning may also be used to gain additional information from one imaging modality based on another imaging modality. For example, O’Brien *et al.*^124^ trained a deep learning network to detect ischemic scar in the left ventricles using a dataset of 200 LGE-CMR and showed that this network can automatically detect scar in routine cardiac computed tomography angiography; similar approaches might be used for detecting scar tissue from computed tomography in the atria.

Predicting whether AF will recur following a specific ablation therapy from pre-ablation imaging metrics may help improve therapy selection. In a pioneering study, Shade *et al.*^125^ used machine learning and mechanistic simulations to predict likelihood of AF recurrence following pulmonary vein isolation using a cohort of 32 paroxysmal AF patients.

Future studies are necessary to assess the effects of different treatment approaches on these relationships and extend these approaches to predict the time of AF recurrence. In these studies, the techniques already developed will need to be extended to different patient groups, including persistent AF patients, and different ablation approaches.

## Conclusions

There are numerous applications of multi-modality imaging for informing treatment strategies for AF. We envisage that new developments in imaging technology and image analysis software will advance the field, improve understanding of the mechanisms underlying AF, and improve safety and precision of ablation therapy. We also propose that approaches that define patient-specific ablation lesion sets tailored to electroanatomic/imaging data and that use machine learning techniques to predict future arrhythmias to inform the choice of ablation approach have the potential to significantly improve catheter ablation outcome.

As discussed, recent technological advances have resulted in a vast array of novel approaches to patient selection, substrate assessment and ablation strategy selection. A critical requirement for any such novel approach prior to adoption is the demonstration in prospective clinical trials of safety and an improvement in patient outcomes resulting from their use. Furthermore, the demonstration of successful implementation outside highly specialized and expert centres is a prerequisite for more generalized use. Meeting these requirements to facilitate widespread clinical uptake is a key, but exciting, challenge which must be embraced in the coming years.

**Conflict of interest:** M.O. has received research support and honoraria from Biosense Webster and has received consultation fees from Medtronic, Biosense Webster, St. Jude/Abbott, and Siemens. S.E.W. has received research support from Biosense Webster, EPD Solutions and consulting fees from Imricor Medical Systems. S.A.N. has received research support from Siemens, Phillips, Abbott, EBR systems, and Pfizer. The remaining authors have no disclosures to report.

### Funding

CR is funded by a Medical Research Council Skills Development Fellowship (MR/S015086/1). SW is supported by the British Heart Foundation, through a fellowship (FS/20/26/34952) and project grant (PG/19/44/34368). SN acknowledges support from the UK Engineering and Physical Sciences Research Council (EP/P01268X/1), the British Heart Foundation (grant nos. PG/15/91/31812, PG/13/37/30280, SP/18/6/33805), US National Institutes of Health (grant no. NIH R01-HL152256) and European Research Council (grant no. ERC PREDICT-HF 864055). All authors acknowledge Kings Health Partners London National Institute for Health Research (NIHR) Biomedical Research Centre and the Wellcome/EPSRC Centre for Medical Engineering (WT 203148/Z/16/Z).
